# Inhibiting PI3K/Akt-Signaling Pathway Improves Neurobehavior Changes in Anti-NMDAR Encephalitis Mice by Ameliorating Blood–Brain Barrier Disruption and Neuronal Damage

**DOI:** 10.1007/s10571-023-01371-3

**Published:** 2023-06-14

**Authors:** Zhuowei Gong, Dayuan Lao, Yu Wu, Taiyan Li, Sirao Lv, Xuean Mo, Wen Huang

**Affiliations:** 1 Department of Neurology, The First Affiliated Hospital of Guangxi Medical University, #6 Shuangyong Road, Nanning, 530021 Guangxi People’s Republic of China; 2 University of Pittsburgh School of Medicine, Pittsburgh, PA USA

**Keywords:** Anti-NMDAR encephalitis, Blood–brain barrier, Tight junction protein, PI3K/Akt-signaling pathway, Neurobehavior change

## Abstract

The disruption of the blood–brain barrier (BBB) is hypothesized to be involved in the progression of anti-*N*-methyl-d-aspartate receptor (NMDAR) encephalitis, but its mechanism is still unclear. Recently, the phosphatidylinositol 3-kinase (PI3K)/threonine kinase (Akt) pathway is involved in the regulation of the BBB in various diseases. This study is aimed to investigate the mechanism of BBB damage and neurobehavior changes in anti-NMDAR encephalitis mice. Female C57BL/6J mice were actively immunized to establish an anti-NMDAR encephalitis mouse model and evaluate the neurobehavior changes of mice. To study its potential mechanism, LY294002 (PI3K inhibitor, 8 mg/kg) and Recilisib (PI3K agonist, 10 mg/kg) were treated by intraperitoneal injection, respectively. Anti-NMDAR encephalitis mice showed neurological deficits, increased BBB permeability, open endothelial tight junctions (TJs), and decreased expression of TJ-related proteins zonula occludens (ZO)-1 and Claudin-5. However, administration of PI3K inhibitor significantly reduced the expression of p-PI3K and p-Akt, improved neurobehavior function, decreased BBB permeability, and upregulated the expressions of ZO-1 and Claudin-5. Furthermore, PI3K inhibition reversed the decline of NMDAR NR1 in the membranes of hippocampal neurons, which reduced the loss of neuron-specific nucleoprotein (NeuN) and microtubule-associated protein 2 (MAP2). In contrast, administration of the PI3K agonist Recilisib showed a tendency to exacerbate BBB breakdown and neurological deficits. Our results showed that the activation of PI3K/Akt, along with the changes in TJ-related proteins ZO-1 and Claudin-5, may be closely related to BBB damage and neurobehavior changes in anti-NMDAR encephalitis mice. PI3K inhibition attenuates BBB disruption and neuronal damage in mice, thereby improving neurobehavior.

**Supplementary Information:**

The online version contains supplementary material available at 10.1007/s10571-023-01371-3.

## Introduction

Autoimmune encephalitis (AE) is a neuroinflammatory disease mediated by antibodies against neuronal or synaptic proteins (Dalmau et al. 2011). One of the most common types of AE is anti-*N*-methyl-d-aspartate receptor (NMDAR) encephalitis. It is characterized by the internalization and degradation of NMDAR in neurons, especially hippocampal neurons, induced by anti-NMDAR NR1 subunit antibodies. This results in a decrease in the density and function of NMDAR on the synaptic surface, leading to neuronal functional impairment (Jézéquel et al. 2017). NMDAR is an ionotropic glutamate receptor, which is expressed in the postsynaptic membrane of excitatory synapses of neurons in the central nervous system (CNS). NMDAR is essential for learning, memory, cognition, and other advanced neural activities (Cull-Candy and Leszkiewicz 2004), of which NR1 is an essential subunit for neurogenesis and survival (Forrest et al. 1994). The clinical manifestations of anti-NMDAR encephalitis are diverse, such as abnormal mental behavior or cognitive dysfunction, seizures, movement disorders, speech disorders, memory decline, autonomic dysfunction, and decreased level of consciousness (Dalmau and Graus 2018). Currently, the drugs used for the treatment of anti-NMDAR encephalitis are not effective enough to improve the recovery of patients. Some patients face severe mental or cognitive impairment, memory impairment, refractory epilepsy, and even death (Dalmau et al. 2008). However, the exact pathophysiological mechanism of anti-NMDAR encephalitis remains unclear. It is generally believed that the triggers of anti-NMDAR encephalitis are viruses and tumors, which may disrupt the blood–brain barrier (BBB) through immune and inflammatory responses. Anti-NMDAR antibodies and lymphocytes enter the CNS through the destroyed BBB. On one hand, these antibodies directly bind to NMDARs; on the other hand, lymphocytes entering the CNS further differentiate into mature plasma cells to produce antibodies, thereby mediating neuronal damage and cell death in the brain (Dalmau and Graus 2018). Hammer et al. found that after injecting anti-NMDAR antibodies purified from patient serum into the tail vein of mice, healthy wild-type mice with intact BBB could effectively prevent anti-NMDAR antibodies from entering the brain without any symptoms; while BBB-deficient APOE−/− mice were unable to prevent the antibodies from entering the CNS, resulting in symptoms such as decreased spontaneous movement (Hammer et al. 2014). Studies have also found that the integrity of the structure and function of the BBB is closely related to the progression of multiple nervous system diseases such as ischemic stroke, traumatic brain injury, multiple sclerosis, and Alzheimer’s disease (Obermeier et al. 2013; Sweeney et al. 2018, 2019). This may indicate that BBB integrity plays a role in anti-NMDAR encephalitis. The disruption of the BBB may be involved in the occurrence and development of anti-NMDAR encephalitis immune brain injury.

The BBB is a highly selective and dynamic barrier between the blood and the brain, which acts as a key homeostatic regulator to promote neuronal survival by restricting the entry of most circulating toxins, blood proteins, microorganisms, and peripheral immune cells into the brain (Sweeney et al. 2019). Therefore, the integrity of the BBB is the key link to protect the CNS from damage. The BBB is composed of cerebrovascular endothelial cells, intercellular tight junctions (TJs), the basement membrane, pericytes, and astrocyte foot processes. The low-permeability part of the BBB is a TJ complex composed of various proteins, mainly including transmembrane proteins such as claudins, occludins, junctional adhesion molecules (JAMs), zonula occludens (ZO) proteins, cytoplasmic attachment proteins, etc. The expression and redistribution of these proteins play an important role in maintaining BBB integrity (Erickson and Banks 2018). Among them, ZO-1 and Claudin-5 are representative proteins of the TJ within the BBB, which can be used as biomarkers of BBB damage in a variety of CNS diseases (Jasiak-Zatońska et al. 2022; Olsson et al. 2021). Some studies also believe that the abnormality of ZO-1 and Claudin-5 can affect the formation of TJs between cells. This offers a new pharmacological target to change the permeability of the BBB (Hashimoto et al. 2021; Wolburg and Lippoldt 2002). Therefore, preserving or restoring the function of the BBB to limit the infiltration of immune cells and autoantibodies into the CNS is expected to become a new therapeutic strategy in the future.

Despite the importance of the BBB, the maintenance and induction factors of its structure and function remain unclear. Some cytoplasmic signaling molecules, such as phosphoinositide-3-kinase (PI3K) have been localized in the TJ complex and may regulate its structure and function (González-Mariscal et al. 2008). PI3K is activated by many receptors and intracellular signaling molecules, promoting the phosphorylation of serine/threonine kinase (AKT) and inducing metabolism, growth, proliferation, and other cellular functions (Hemmings and Restuccia 2012). It has been reported that PI3K/Akt signaling is involved in peripheral NMDAR-mediated activation of vascular nitric oxide and ROS, which ultimately leads to increased calcium influx (Xie et al. 2018). Several studies have shown that the PI3K/Akt pathway can affect brain development and induce various neurological diseases by regulating neuronal cell proliferation and differentiation, autophagy and apoptosis, synaptic plasticity, and circuits (Hou and Klann 2004; Wu et al. 2020). Furthermore, activation of the PI3K/Akt pathway induces TJ opening in human brain microvascular endothelial cells (Wang et al. 2011). The PI3K/Akt pathway is also closely related to TJ proteins. Studies have shown vascular endothelial growth factors (VEGFs) downregulate the expression of Claudin-5 through the PI3K/Akt/Snail2 signal pathway in human cerebral vascular endothelial cells and human aortic endothelial cells (Laakkonen et al. 2017). In addition, granulocyte–macrophage colony-stimulating factor (GM-CSF) inhibits the expression of ZO-1 through PI3K/Akt/miR-96/ERG (erythroblast transformation-specific transcription factor) in cerebral microvascular endothelial cells (Zhang et al. 2018). PI3K inhibitors can increase endothelial function and can be used to treat a variety of chronic inflammatory diseases and neurodegenerative diseases (Cain et al. 2010). LY294002 is a potent PI3K inhibitor. Studies have shown that LY294002 can prevent NMDA-induced excitotoxicity-related retinal diseases by improving retinal neuron and blood vessel damage in rats (Ueda et al. 2013). Microtubule-associated protein 2 (MAP-2) is an important indicator for evaluating neuronal plasticity, whereas neuron-specific nucleoprotein (NeuN) is used as a neuronal marker (Johnson and Jope 1992; Wolf et al. 1996). Both can be used to evaluate the immune damage of neurons in the brain of mice with anti-NMDAR encephalitis.

**Fig. 1:**
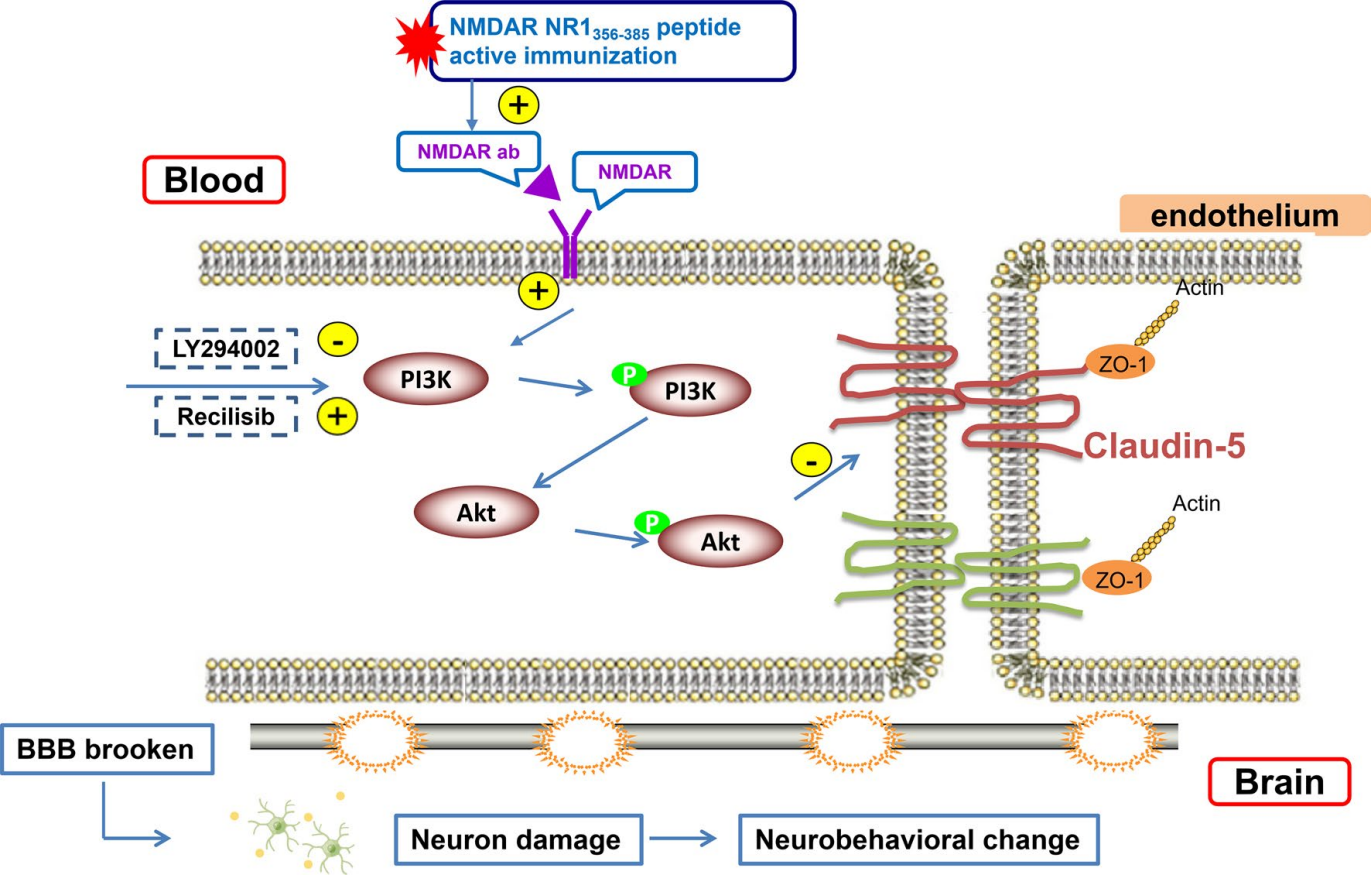
Schematic diagram of different site interventions of PI3K/Akt signal pathway

Mice were actively immunized with NMDAR NR1_356–385_ peptide in this study and explored the role of the PI3K/Akt signaling pathway on BBB destruction and neuronal damage. The schematic diagram of PI3K/Akt signal pathway intervention is shown in Fig. 1. Our new observations suggest that PI3K inhibition can increase the expression of TJ-related proteins ZO-1 and Claudin-5, reduce BBB permeability, reduce NMDAR internalization and neuronal loss, and improve neurological function in mice with anti-NMDAR encephalitis by inhibiting PI3K/Akt phosphorylation.

## Materials and Methods

### Animals and Treatment

8-week-old female C57BL/6J mice (18–22 g) were purchased from the Animal Experiment Center of Guangxi Medical University (Guangxi, China). The female mice were housed in standard plastic cages (6 mice in each cage) and a 12-h light/12-h dark cycle. Food and drinking water were available ad libitum. All experiments complied with the requirements of the National Institutes of Health Guidelines for the Care and Use of Laboratory Animals. All procedures and protocols have been approved by the Animal Experiment Ethics Committee of Guangxi Medical University (Certificate Numbers: 202010029).

The anti-NMDAR encephalitis mouse model that was used is the C57BL/6J mice (8 weeks old, female). The model was established by peptide active immunization (Ding et al. 2021). The mice were immunized with extracellular peptides containing NMDAR NR1_356–385_ (P210812-XT880902), which were emulsified in complete Freund's adjuvant (CFA) containing *Mycobacterium tuberculosis* H37Ra (4 mg/mL). The final peptide concentration was 1 mg/mL. The mice were subcutaneously immunized with 200 μg of the polypeptide mixture, of which the mice in the control group were subcutaneously immunized with a mixture of equal volumes of CFA and phosphate-buffered saline (PBS). A booster injection was given 4 weeks after the immunization. All mice were intraperitoneally injected with 200 ng pertussis toxin on the day of immunization and 48 h after immunization.

Animals were randomly divided into 4 groups: control group, model group, model + LY294002 group, and model + Recilisib group. Mice in the treatment group were intraperitoneally administered LY294002 (HY-10108, PI3K inhibitor, 8 mg/kg) or Recilisib (HY-101625, PI3K agonist, 10 mg/kg) every day. LY294002 and Recilisib were from MedChemExpress, USA, and the above treatments were given once a day for 7 consecutive days. All PI3K inhibitors and agonists were dissolved in dimethyl sulfoxide/saline (1:10). The same amount of sterile solvent was injected intraperitoneally into the control group and model group.

### Assessment of Neurological Deficits

#### Short-Term Neurological Function Evaluation

Observation of neurological function started on the day of modeling in the model group, model + LY294002 group, and model + Recilisib group. On the 1st, 7th, and 14th days, two researchers who were blind to the experiment adopted the modified neurological severity score (mNSS) method (Asadi et al. 2018) to evaluate the degree of short-term neurological deficit. The mNSS neurological deficit score is recorded from 0 (normal) to 18 (maximum deficit). A higher score indicated more serious damage.

#### Long-Term Neurological Function Assessment

The Morris water maze (Xie et al. 2018) was used to evaluate the long-term neurological deficit. A group of random starting positions was used to place the mice. The platform was located 1 cm underwater in the center of the target quadrant and the monitoring time was the 60 s. The time taken by the mice to board the platform for the first time was recorded. The average value of 4 times was taken as the escape latency. The mice were guided to stay on the platform for 5 s for 5 days of acquired training. On the 6th day, the exploration test was performed, the platform was removed, and the computer tracking system (Smart v3.0) recorded the number of times that the mice crossed the original platform position within 60 s. The travel distance and time in the target quadrant were used as the detection index of spatial memory.

### Evaluation of BBB Damage

#### BBB Permeability Test

The BBB permeability of mice was detected by the sodium fluorescein method. The mice were intraperitoneally injected with 200 μL sodium fluorescein solution (F299344, 0.1 g/mL, dissolved in sterile phosphate-buffered saline) and the solution was circulated for 30 min. The mice were anesthetized with isoflurane in oxygen. The blood was collected through the cardiac puncture, centrifuged for segregating plasma, and an equal volume of 15% TCA was added and mixed. The mice were perfused with 30 mL PBS through the left ventricle, the brains were decapitated and weighed, and 7.5% trichloroacetic acid (TCA) was added to the homogenate. After centrifugation at 10,000 r for 30 min, the supernatant (500 μL) was neutralized with 125 μL NaOH (5 mol/L). The absorbance values at 485 nm and 530 nm of the supernatant were detected with a spectrophotometer. BBB permeability was calculated as the ratio of sodium fluorescein in the brain to plasma.

#### TEM Assay

Mice were transcardially perfused with 2% paraformaldehyde. The brains were removed at room temperature and 1 mm^3^ brain tissues were fixed in fixative, which was prepared with 3% glutaraldehyde (pH 7.2–7.4) at 4 °C for 2 h. After washing, the tissues were then fixed in 1% Osmic acid at 4 °C for 2 h. After dehydrating in graded ethanol, penetrating, and epoxy resin embedding, Ultrathin section (60–80 nm) were prepared and stained with lead citrate. They were then observed under a transmission electron microscope (HT7800, HITACHI) and images were collected for analysis.

### Expression of PI3K/Akt, NMDAR NR1, and Tight Junction Protein in Mice with Anti-NMDAR Encephalitis

#### Western Blot Analysis

The membrane protein extraction kit (P0033, Beyotime Biotechnologies) was used to extract membrane protein from the hippocampus. In addition, total protein was extracted with RIPA lysis buffer, which contains phosphatase and protease inhibitors. The prepared protein samples were separated by 6% to 10% Tris–HCl Ready SDS–polyacrylamide gel, then transferred to PVDF membrane and incubated overnight at 4 °C with the corresponding antibody. Claudin-5 antibodies were purchased from Thermo Fisher Scientific (PA5-99415, Boston, USA). NMDAR NR1 antibodies were purchased from Abcam (ab109182, Cambridge, UK). Anti-zonula occludens-1 (ZO-1), neuron-specific nuclear protein (NeuN), microtubule-associated protein-2 (MAP-2), β-actin, anti-total-PI3K (T-PI3K), phospho-PI3K (p-PI3K), anti-total-Akt (T-Akt), and phospho-Akt (p-Akt) antibodies were from Cell Signaling Technology (#8193, #12943, #8707, #3700, #4257, #4228, #4691, #4060, Respectively, Boston, USA). GAPDH antibodies were purchased from Proteintech Group (10494-1-AP, Chicago, USA). After the membrane was washed, goat anti-rabbit fluorescent secondary antibody (1: 15,000, SA5-35571, Thermo Fisher Scientific, Boston, USA) was added and incubated at room temperature for 1 h. Bands were observed in the Odyssey infrared Imaging system (LI-COR Biosciences) and analyzed semi-quantitatively by ImageJ software (National Institutes of Health, Bethesda, USA). The limitation of the study was the lack of testing of the specificity of the primary antibodies.

#### Quantitative Real-Time Polymerase Chain Reaction (qRT-PCR)

For quantitative real-time polymerase chain reaction (qRT-PCR), TRIzol reagent (RR036A, Takara, Dalian, China) was used to extract total RNA from brain tissue. The mRNA levels of target genes were normalized by endogenous control GAPDH levels. The expression levels of ZO-1, Claudin-5, and NMDAR NR1 mRNA were calculated by the formula 2^−ΔΔCt^. Primers were designed according to previous research (Mao et al. 2022; Yu et al. 2022). The primer sequence of NMDAR NR1 is as follows: F: AGAATGTGACTCCCGCAGCAATG, R: GGGCATCCTTGTGTCGCTTGTAG.

#### Immunofluorescence Staining

The mouse brain sections were fixed with 4% paraformaldehyde. After permeabilization and blocking, samples were incubated overnight at 4 °C with primary antibodies to the following proteins: ZO-1 (#8193, Cell Signaling Technology, USA, 1:200), Claudin-5 (PA5-99415, Thermo, USA, 1:300), and CD31 (GB11063-2, Servicebio, China, 1:200). Subsequently, samples were incubated with appropriate fluorescently conjugated secondary antibodies for 1 h at room temperature in the dark. DAPI (G1012, Servicebio, 1:500) staining was used to reveal the nuclei. Images were examined using the 20× objective under a fluorescence microscope (Nikon E100, Japan) and equipped with a fluorescence scanner (Pannoramic 250FLASH, 3DHISTECH, Hungary). The images obtained in Tiff format were adjusted for brightness and contrast using the software Caseviewer c.v 2.3, diagnostic instrument.

### Determination of Neuron Loss

The expression of NeuN in the brain was analyzed by Western blotting and immunohistochemistry. The Western blotting method was as previously described. For immunohistochemistry, the position containing the ventral hippocampus was selected and cut into 10 μm thick coronal sections. The sections were incubated overnight at 4 °C with mouse anti-NeuN antibody (#12943, Cell Signaling Technology, 1:500). After washing, the samples were incubated with the appropriate HRP-conjugated secondary antibody (GB23303, Servicebio, 1:400) for 1 h at room temperature. Microscopic examination (BX53, Olympus), image acquisition, and image analysis (ImageJ software, Bethesda, USA) were subsequently performed.

### Statistical Analysis

All data were statistically analyzed by SPSS 23.0 (SPSS, Chicago, IL, USA). The data were expressed as mean ± standard deviation. One-way ANOVA was used to compare the differences between groups. Graphpad Prism 7.0 Software (San Diego, CA, USA) was used to draw the histogram. All experiments were repeated at least three times. *P* < 0.05 is statistically significant. The authors were blind to the experimental protocol or lacked statistical calculations when conducting experiments.

## Results

### Elevated Serum and Cerebrospinal Fluid NMDAR NR1 Antibodies in Model Mice

**Fig. 2:**
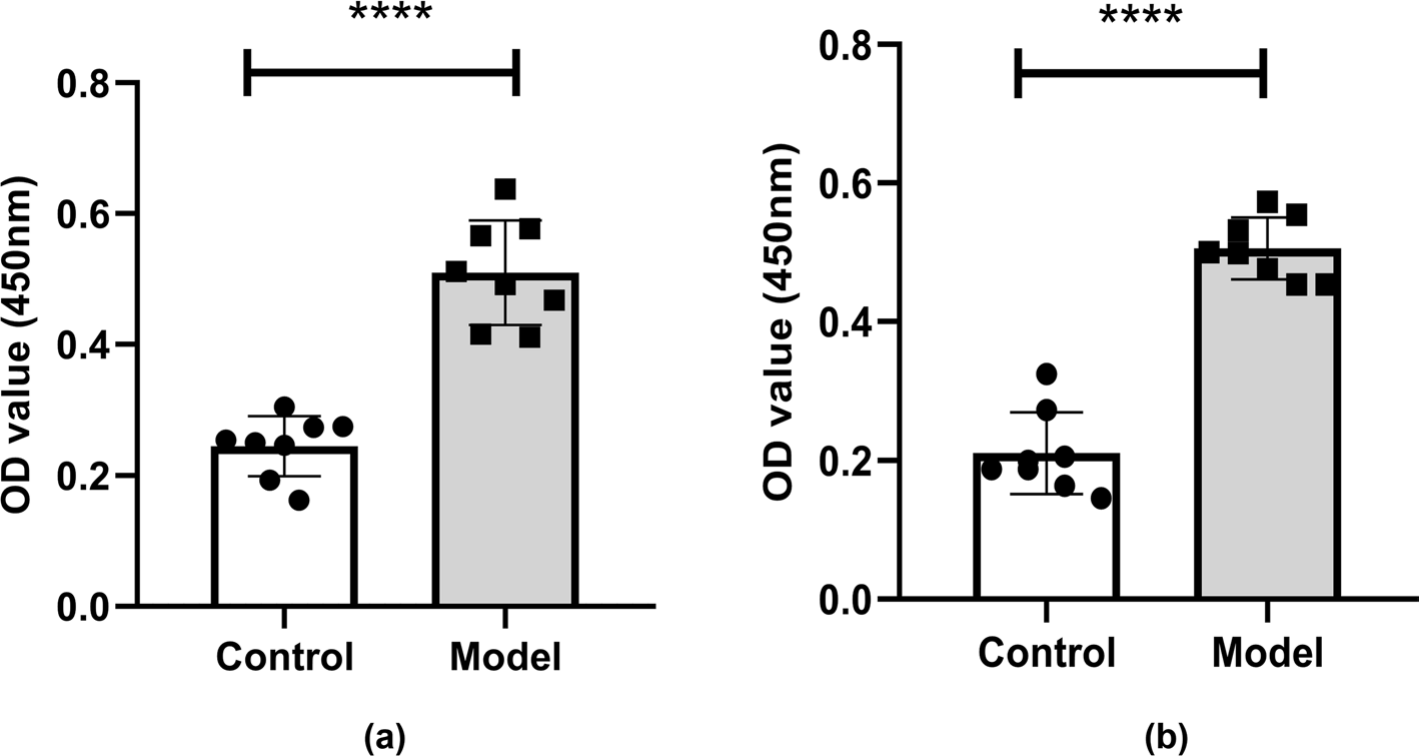
Anti-NMDAR NR1 IgG levels in cerebrospinal fluid (**a**) and serum (**b**) of mice at the 6th week of modeling. The levels of cerebrospinal fluid and serum anti-NMDAR NR1 IgG in the model group were significantly higher than those in the control group. The differences were statistically significant. Values are expressed as mean ± SD (*n* = 8, technical replicates = 2). ****P < 0.0001, versus the control group

At the 6th week of immunization, the cerebrospinal fluid (CSF) and orbital blood of the mice were collected. The anti-NMDAR NR1 antibody levels in the CSF and serum were detected with an anti-NMDAR NR1 antibody kit. As shown in Fig. 2, the levels of anti-NMDAR antibodies in the CSF (a) and serum (b) of the mice in the model group were significantly increased in comparison to the control group. According to the kit instructions, the antibody positive threshold = negative standard OD value + 0.25. If the OD value of the test sample is greater than the positive threshold, it can be determined that the anti-NMDAR antibody is positive. The results showed that the OD value of CSF was 0.25 ± 0.046 in the control group and 0.51 ± 0.080 in the model group. The CSF negative threshold was 0.058 and the positive threshold was 0.308. It can be determined that the anti-NMDAR IgG antibody of CSF in the model group was positive (Fig. 2a). The OD value of serum was 0.21 ± 0.059 in the control group and 0.51 ± 0.045 in the model group. The serum negative threshold was 0.098 and the positive threshold was 0.348. It can be determined that the anti-NMDAR IgG antibody of serum in the model group was positive (Fig. 2b).

### The Effect of PI3K on the Neurobehavior and Cognition of Anti-NMDAR Encephalitis Mice

**Fig. 3:**
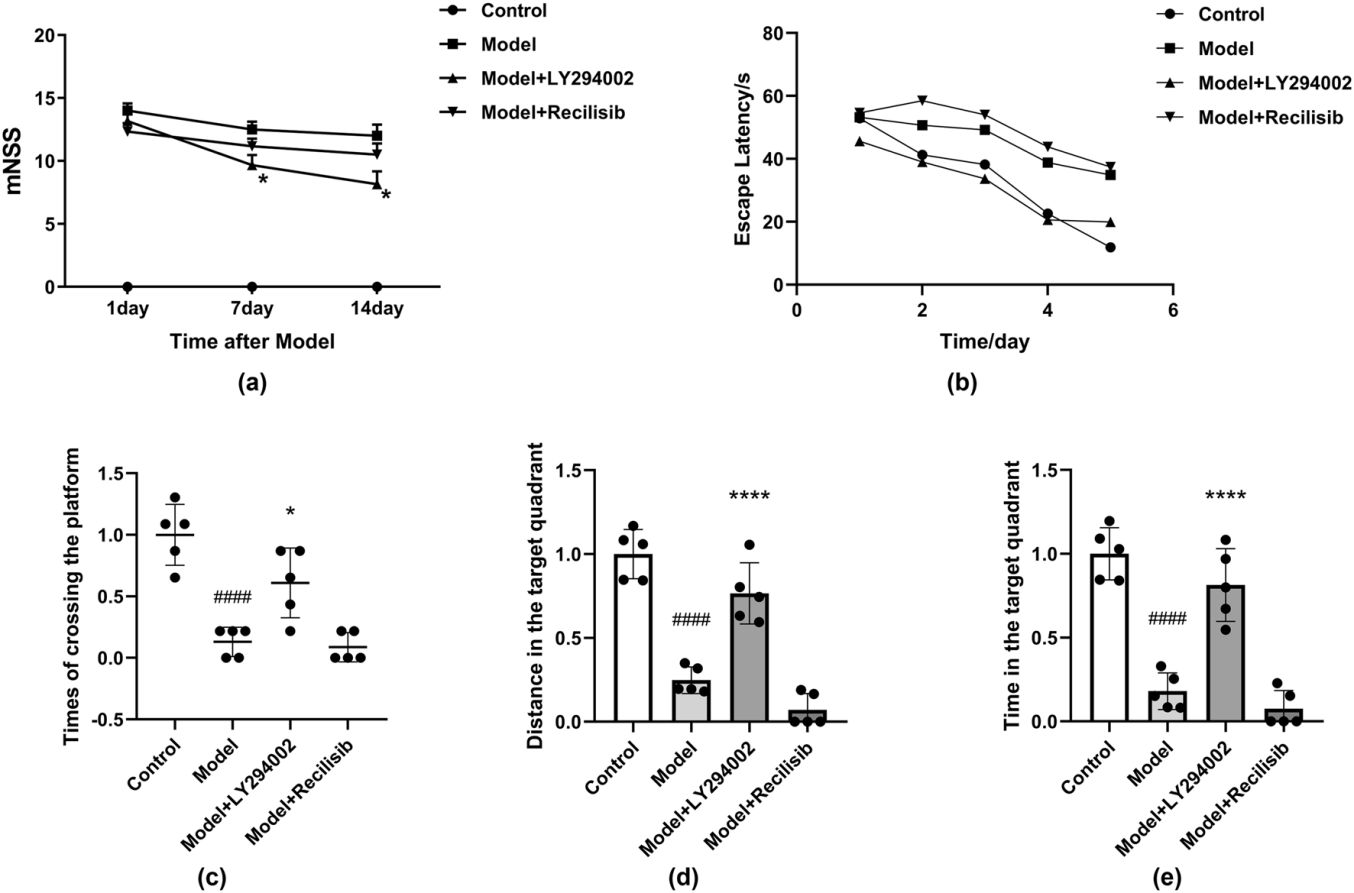
PI3K inhibition improving neurological deficits in anti-NMDAR encephalitis mice. The short-term neurological function was assessed by the modified neurological severity score (mNSS) method. Mice in the control group and model group were intraperitoneally injected with vehicle, while those in the treatment group were intraperitoneally injected with LY294002 (PI3K inhibitor, 8 mg/kg) or Recilisib (PI3K agonist, 10 mg/kg) once a day for 1 week. After model establishment, scores increased at different time points, but significantly improved after LY294002 infusion. However, there was no significant difference in mNSS scores between the Recilisib group and the model group (**a**). LY294002 attenuates memory deficits and improves long-term neurological function in mice with anti-NMDAR encephalitis. Administration of LY294002 improved water maze performance in the model mice. The escape latency of the mice during the acquired training period (**b**). The times of crossing the platform during the exploration training period (**c**). The distance in the target quadrant during the exploration training period (**d**). The time in the target quadrant during the exploration training period (**e**). Values are expressed as mean ± SD (*n* = 4). ^####^*P* < 0.0001, versus the control group; *P < 0.05, ****P < 0.0001, versus the model group

Six weeks after immunization, the mNSS method was performed on the mice in each group on the 1st, 7th, and 14th day (Fig. 3a). The control group had no neurological deficits and the score was 0. The scores of the mice in the model group were significantly higher, indicating severe neurological impairment. The neurological score of the model + LY294002 (PI3K inhibition) group was lower than that of the model group, but still higher than that of the control group. There was no significant difference in the mNSS score after Recilisib (PI3K agonist) treatment compared with the model group. PI3K inhibition reduces short-term neurological damage in mice with anti-NMDAR encephalitis.

In the Morris water maze test, there was no significant difference in the escape latency between the groups on the first day, but the escape latency of the model group was significantly higher than that of the control group on the second day (Fig. 3b). On the sixth day of the exploration test, the number of times that the mice crossed the platform, the distance, and time in the target quadrant for the model group were reduced compared to the control group. However, these results were significantly increased in the model + LY294002 group. In contrast, the PI3K agonist Recilisib tended to further aggravate the neurological deficit (Fig. 3c–e), but the difference was not statistically significant. Memory deficits were significantly improved in mice treated with LY294002.

### PI3K Inhibition Prevented the Destruction of Blood–Brain Barrier Integrity in Anti-NMDAR Encephalitis Mice

**Fig. 4:**
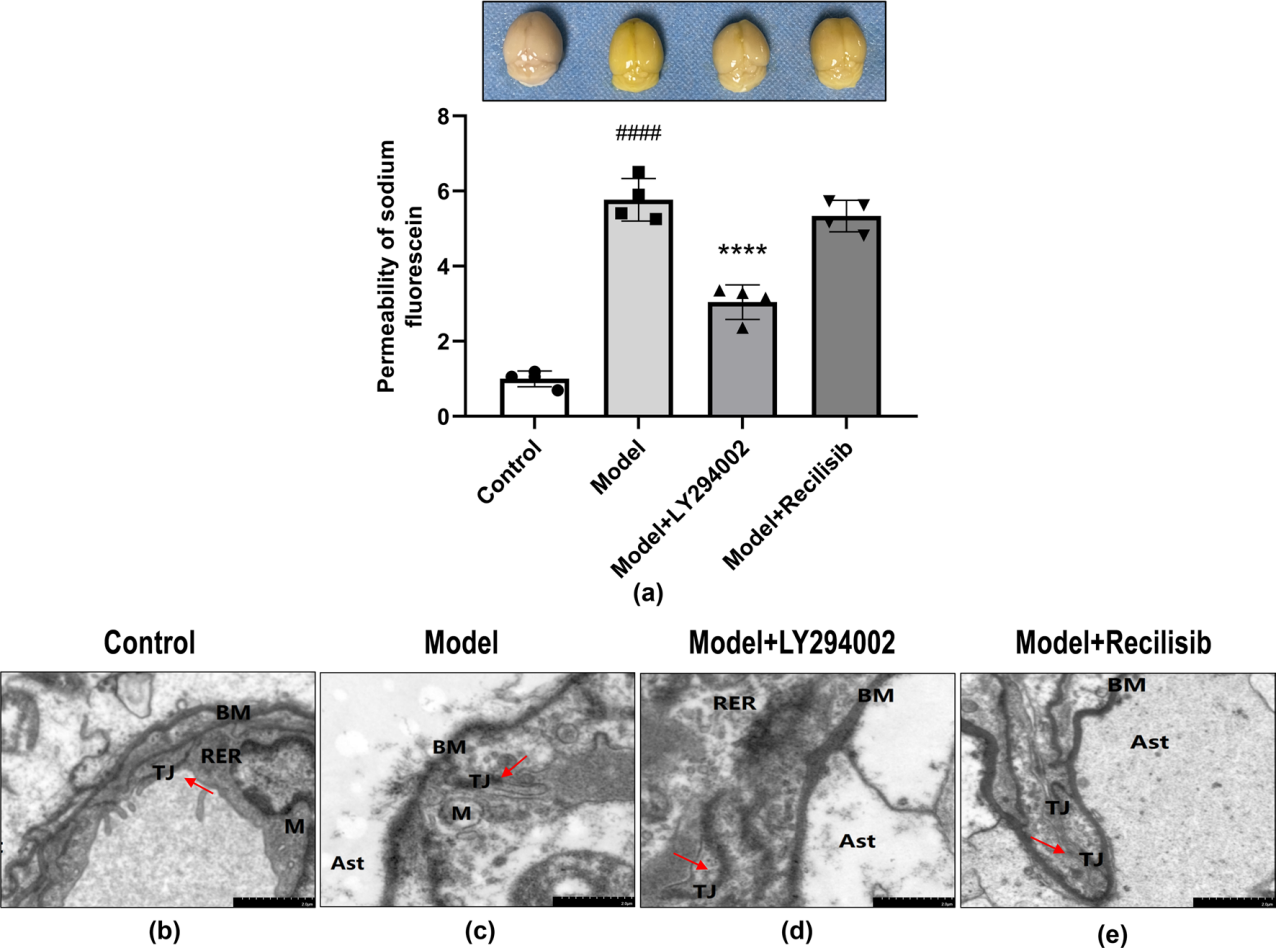
The PI3K inhibition preventing disruption of BBB integrity in anti-NMDAR encephalitis mice. BBB permeability was assessed within 24 h after 1 week of dosing using sodium fluorescein as a marker (**a**), which was macroscopically stained with highly disrupted BBB in yellow (upper panel). Quantification of the effect on BBB leakage rate is shown in the bar graph (lower panel). Results are mean ± SD (*n* = 4). ^####^*P* < 0.0001, versus the control group; ****P < 0.0001, versus the model group. The degree of damage to the tight junctions (TJs) of brain microvascular endothelial cells was observed using a transmission electron microscope (**b–e**). Compared with the control group, most of the TJs were severely damaged in the model group, the number of TJs between cells was significantly reduced, and the dense area was blurred and discontinuous (red arrow). The local structure of the basement membrane (BM) is blurred, fragmented, and discontinuous. The number of mitochondria (M) is small and the local membrane of the rough endoplasmic reticulum (RER) is damaged. Astrocyte (Ast) showed severe edema, matrix dissolution, and organelle disintegration. Compared with the model group, the PI3K inhibitor LY294002 can significantly improve the ultrastructural changes of the BBB in anti-NMDAR encephalitis mice. The PI3K agonist Recilisib showed no significant difference versus the model group

To further verify the relationship between the changes of the neurobehavior function and BBB damage in mice, the BBB permeability of anti-NMDAR encephalitis mice was measured by calculating the content of sodium fluorescein entering the brain parenchyma. Compared with the control group, the leakage of sodium fluorescein in the model group was significantly increased, suggesting that the permeability of the BBB was increased and the integrity of the BBB was destroyed. In cases where BBB integrity is severely compromised, increased levels of sodium fluorescein in brain tissue will result in the yellowing of the brain (Fig. 4a, upper panel). Notably, the PI3K inhibitor LY294002 attenuated BBB hyperpermeability in anti-NMDAR encephalitis mice, as quantified in the lower panel of Fig. 4a. In contrast, the PI3K agonist Recilisib further exacerbated the damage of BBB integrity in anti-NMDAR encephalitis mice.

Under the transmission electron microscope, there was no obvious abnormality in the ultrastructure of the BBB in the control group (Fig. 4b). In the model group, it was observed that the TJs of most brain microvascular endothelial cells were severely damaged, the number of TJs between cells was significantly reduced, and the dense areas were blurred and discontinuous (Fig. 4c). The intervention of PI3K inhibitor LY294002 significantly improved the ultrastructural changes of BBB in anti-NMDAR encephalitis mice (Fig. 4d). Compared with the model group, the damage of TJs in brain microvascular endothelial cells was reduced. The BBB damage after the intervention of PI3K agonist Recilisib was more obvious (Fig. 4e). These results confirmed that the changes of BBB permeability in anti-NMDAR encephalitis mice were closely related to the damage of ultrastructure of TJs formed by endothelial cells, and the PI3K inhibition attenuated the damage of TJ ultrastructure to a certain extent.

### PI3K Intervention Changed the Expression of TJ-Related Proteins ZO-1 and Claudin-5 in Anti-NMDAR Encephalitis Mice

**Fig. 5:**
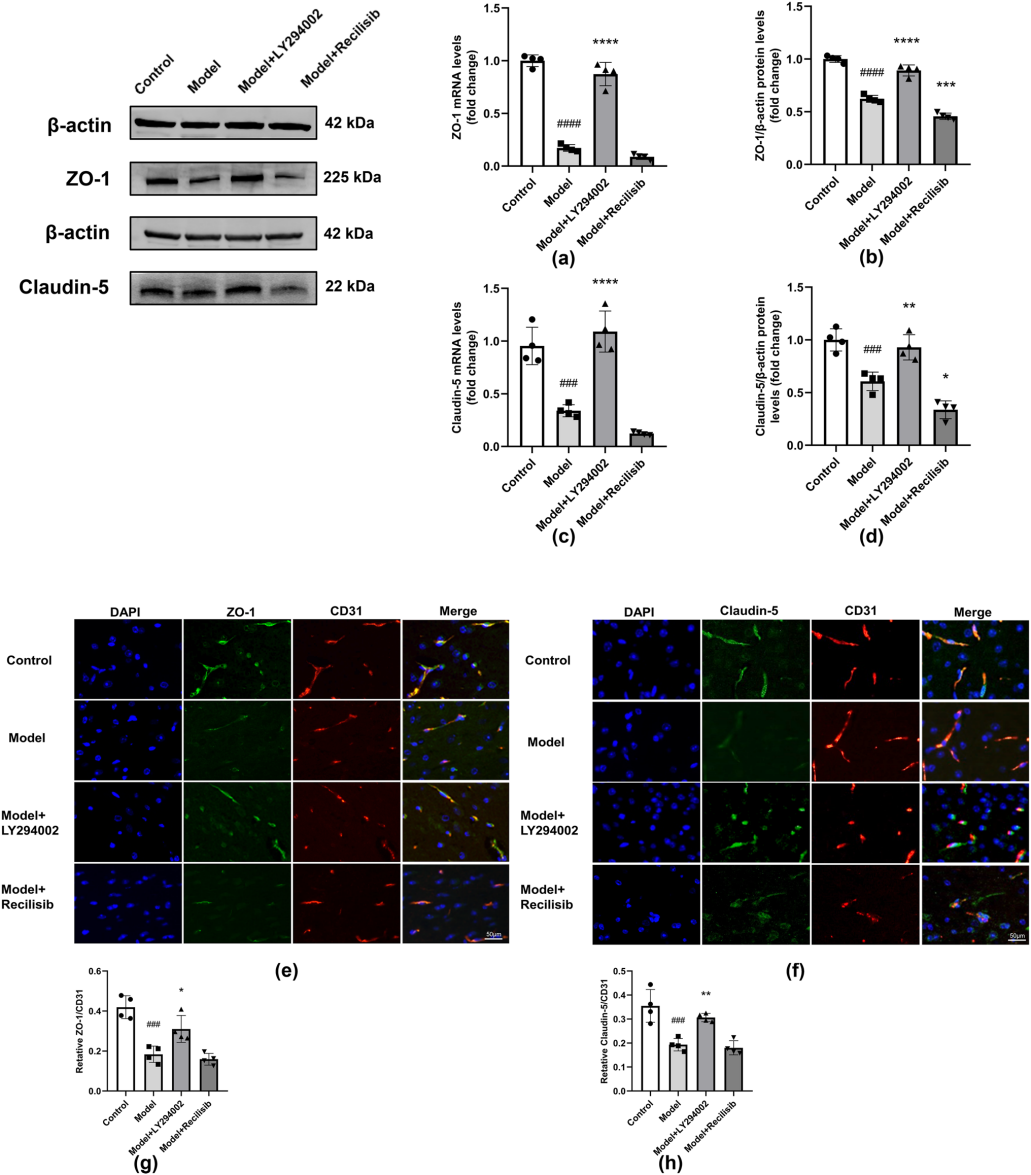
PI3K inhibition hindering the reduction of TJ-related proteins ZO-1 and Claudin-5 in mice with anti-NMDAR encephalitis. The protein and mRNA levels of ZO-1 and Claudin-5 were detected by Western blotting and qRT-PCR, respectively. Compared with the control group, the protein and mRNA levels of ZO-1 and Claudin-5 in the model group decreased; LY294002 reversed this change. Recilisib further decreased the protein and mRNA levels of ZO-1 and Claudin-5 (**a–d**). Representative immunofluorescence images of DAPI (blue)/ZO-1 (green) and Claudin-5 (green)/CD31 (red) colocalization (**e, f**). Scale bar: 50 μm. Quantification of the immunofluorescence intensity of ZO-1 with respect to that of CD31 (**g**). Quantification of the immunofluorescence intensity of Claudin-5 with respect to that of CD31 (**h**). All data are expressed as mean ± SD (*n* = 4, technical replicates = 2). ^###^*P* < 0.001, ^####^*P* < 0.0001, versus the control group; *P < 0.05, **P < 0.01, ***P < 0.001, ****P < 0.0001, versus the model group

The expression levels of the TJ-associated protein ZO1 and the transmembrane TJ protein Claudin-5 were assessed by Western blotting and qRT-PCR. The results showed that the protein and mRNA levels of ZO-1 and Claudin-5 were significantly reduced in the model group, compared with the control group. However, the reduction of ZO-1 and Claudin-5 expression in anti-NMDAR encephalitis mice was blocked after intervention with PI3K inhibitor LY294002, compared with the model group (Fig. 5a–d). The reduction of ZO-1 and Claudin-5 expression was further exacerbated in the PI3K agonist Recilisib group. Based on these results, the expression of ZO-1 and Claudin-5 was further detected by immunofluorescence double-labeled staining. The results showed that the co-localization of ZO-1, Claudin-5, and endothelial marker CD31 was significantly reduced in the model group, compared with the control group. However, the expression levels of these proteins were significantly increased after the intervention of PI3K inhibitor LY294002. In contrast, there was no significant difference between the PI3K agonist Recilisib group and the model group (Fig. 5e–h). These data suggest that PI3K inhibition can regulate the expression of TJ-related proteins ZO-1 and Claudin-5. PI3K inhibition may also maintain the integrity of the BBB after brain injury in mice with anti-NMDAR encephalitis.

### The Effect of Inhibiting PI3K on NMDAR Expression in Hippocampal Neurons of Mice with Anti-NMDAR Encephalitis

**Fig. 6:**
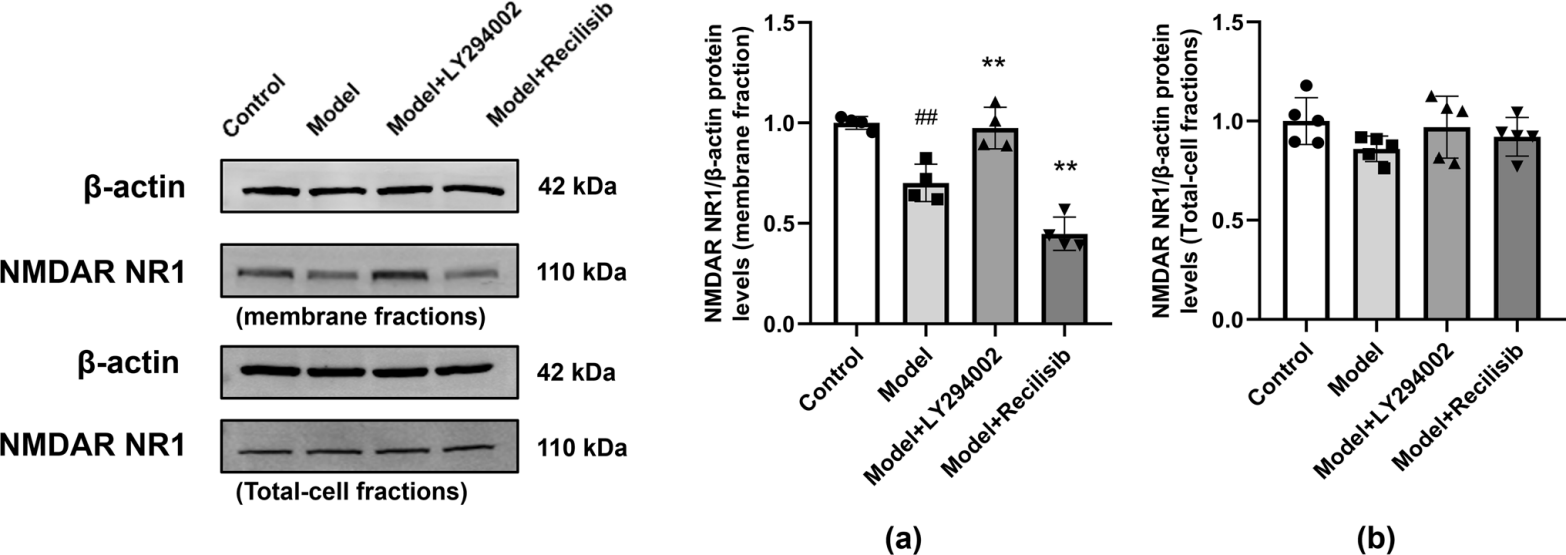
PI3K inhibition changing the expression of NMDAR on the surface of hippocampal neuron membrane in anti-NMDAR encephalitis mice. The expression of NMDAR NR1 in the hippocampal neuron membrane was detected by Western blot. Compared with the control group, the expression of NMDAR NR1 in anti-NMDAR encephalitis mice decreased, and the PI3K inhibitor LY294002 reversed this change. In the agonist group, the expression of NMDAR NR1 protein in the hippocampal neuron membrane was further reduced (**a**). There was no significant difference in the expression of total protein NMDAR NR1 among the groups (**b**). Values are expressed as mean ± SD (*n* = 4). ^##^*P* < 0.01, versus the control group; **P < 0.01, versus the model group

The hippocampal tissues of immunized mice were collected and the membrane proteins were extracted to detect the expression of NMDAR NR1. Western blot showed that the number of hippocampal neuron membranes of NMDAR NR1 in mice was significantly reduced after active immunization, indicating a decrease in NMDAR on the synaptic cell surface (Fig. 6a). Whether NMDAR expression levels were affected by PI3K inhibitors or agonists was further determined and compared with the model group. PI3K inhibitors upregulated the expression of NMDAR NR1, while the agonist group further downregulated it. In addition, there was no significant difference in the expression of total protein NMDAR NR1 among the groups (Fig. 6b).

### The Protective Effect of PI3K Inhibition on Neuron Loss in Anti-NMDAR Encephalitis Mice

**Fig. 7:**
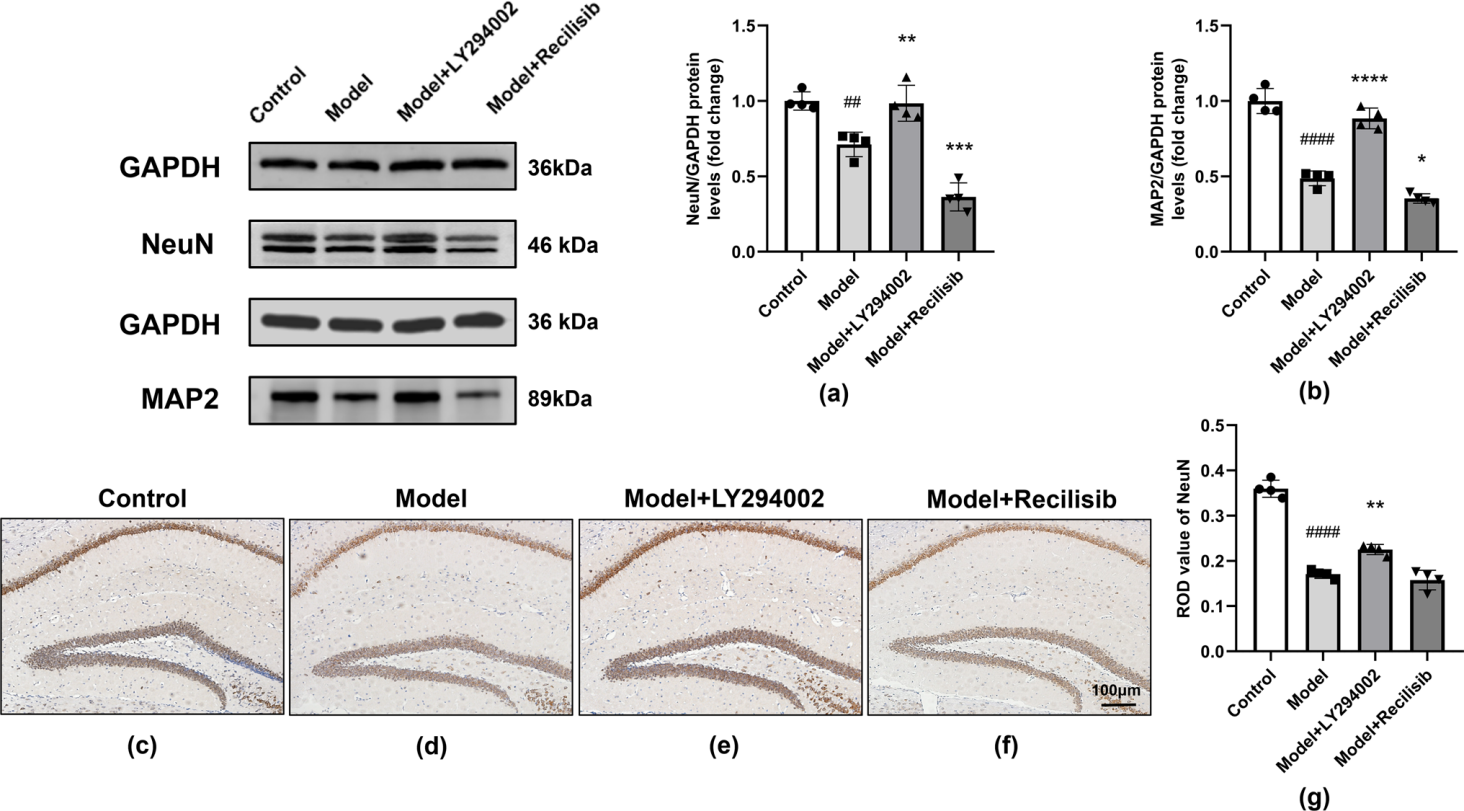
PI3K inhibition improving neuron loss in anti-NMDAR encephalitis mice. The level of NeuN and MAP2 protein was detected by Western blot. Compared with the control group, the level of NeuN and MAP2 protein in the model group decreased. The level of NeuN and MAP2 protein after LY2994002 treatment was higher than that in the model group, while the levels of NeuN and MAP2 protein in the Recilisib treatment group were further decreased (**a, b**). The representative immunohistochemistry (IHC) images of the NeuN-positive cells in the unilateral hippocampus are shown in (**c–f**), and quantification is shown in (**g**). Compared with the control group, the number of NeuN-positive cells in model mice decreased and the treatment of PI3K inhibitor LY2994002 alleviated the loss of neuron cells, while the treatment of PI3K agonist Recilisib showed no significant difference. The scale represents 100 μm. Values are expressed as mean ± SD (*n* = 4). ^##^*P* < 0.01, ^####^*P* < 0.0001, versus the control group; *P < 0.05, **P < 0.01, ***P < 0.001, ****P < 0.0001, versus the model group

Neurons are the main target of immune damage in anti-NMDAR encephalitis. Neuronal damage is thought to underlie the development of cognitive dysfunction, often observed in CNS diseases. Therefore, neuron-specific nuclear protein (NeuN) was used as a specific neuronal marker to assess neuronal loss in anti-NMDAR encephalitis mice and the effect of PI3K inhibition on it, whereas microtubule-associated protein 2 (MAP2) was used to detect neuronal plasticity in brain tissue. Western blotting (Fig. 7a, b) showed that the expression levels of NeuN and MAP-2 were reduced, while PI3K inhibitors significantly upregulated their expression levels. Compared with the model group, the PI3K agonist group further downregulated the expression of NeuN and MAP-2. In addition, immunohistochemistry staining (Fig. 7c–f) showed that the neuronal NeuN protein was positive in the brain tissues of each group. The protein was a brownish-yellow or brown precipitate, distributed in the cytoplasm and capsule. Compared with the control group, the neuronal cell death of mice in the model group increased significantly (Fig. 7d). However, these effects were attenuated after treatment of mice with the PI3K inhibitor LY294002 (Fig. 7e). Compared with the model mice, there was no significant difference in the PI3K agonist Recilisib group (Fig. 7f). Quantification is shown in Fig. 7g. Our data showed that PI3K inhibition significantly improves nerve damage and remodeling in anti-NMDAR encephalitis mice.

### PI3K Inhibition Against the Activation of the PI3K/Akt Signaling in Anti-NMDAR Encephalitis Mice

**Fig. 8:**
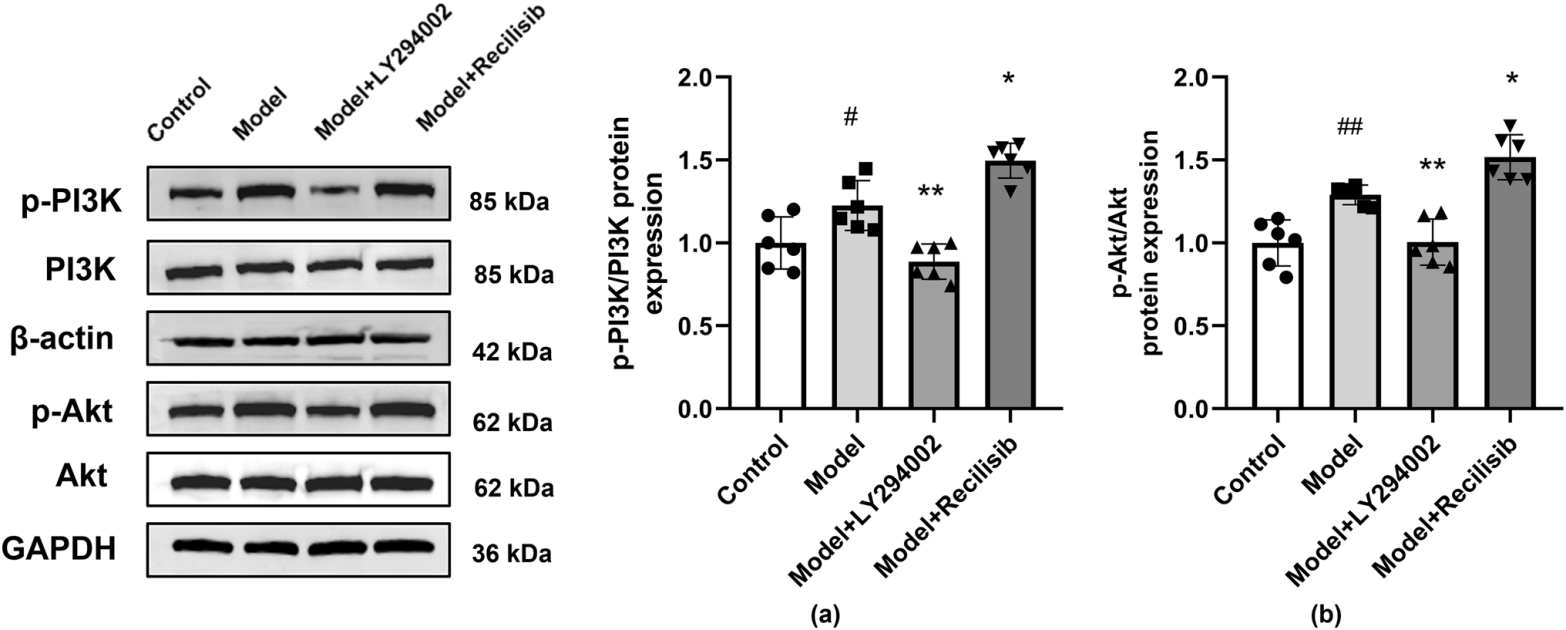
PI3K inhibition hindering the activation of the PI3K/Akt signaling in anti-NMDAR encephalitis mice. The levels of total PI3K, Akt, phosphorylated PI3K(p-PI3K), and phosphorylated Akt(p-Akt) proteins were detected by Western blotting. Compared with the control group, the protein levels of p-PI3K and p-Akt in the model group were significantly increased. Compared with the model group, the LY294002 treatment decreased the protein levels of p-PI3K and p-Akt, while the Recilisib treatment increased these proteins. The quantification of stripes is shown in (**a, b**). Values are expressed as mean ± SD (*n* = 6). ^#^*P* < 0.05, ^##^*P* < 0.01, versus the control group; *P < 0.05, **P < 0.01, versus the model group

PI3K/Akt pathway is involved in regulating TJ protein integrity, synaptic plasticity, along with learning and memory function. Therefore, in order to further prove that the PI3K/Akt pathway is involved in BBB damage and the neurobehavior changes of anti-NMDAR encephalitis mice, the phosphorylation of PI3K and Akt was detected by Western blotting in this study. Compared with the control group, the protein levels of p-PI3K and p-Akt in the model group were significantly higher. Consistent with TJ expression data, the PI3K inhibitor LY294002 hindered the activation of the PI3K/Akt pathway in anti-NMDAR encephalitis mice. However, the PI3K agonist Recilisib enhanced PI3K and Akt phosphorylation in these animals (Fig. 8a, b).

## Discussion

The degree of structural and functional disruption of the BBB is closely related to the occurrence, development, and outcome of various AEs, such as anti-NMDAR encephalitis, limbic encephalitis, and postinfectious basal ganglia encephalitis (BGE). Magnetic resonance imaging (MRI) Gd enhancement can reveal underlying BBB damage or foci of BBB damage in patients with AE (Platt et al. 2017). Therefore, our working hypothesis is that BBB disruption is a critical step in the pathogenesis of anti-NMDAR encephalitis mice. Peripheral inflammation, immune cells, and toxic substances are recruited and infiltrated into the CNS through the disrupted BBB, leading to neurodegeneration. Therefore, drugs that can maintain the integrity of the BBB may have the potential to treat many neurological diseases including AE, Alzheimer's disease, and multiple sclerosis (Sweeney et al. 2019). Studies have shown that damage to TJ proteins may play a role in the pathological process of AE blood–brain barrier disruption and neurovascular injury (Platt et al. 2017). It is worth mentioning that PI3K has been shown to regulate vascular integrity and angiogenesis during development. PI3K also contributes to tumor necrosis factor (TNF)-induced disruption of endothelial barrier function by regulating TJs between endothelial cells (Cain et al. 2010). Multiple studies have shown that the PI3K/Akt signaling pathway is involved in the opening of endothelial TJs in various diseases (Platt et al. 2017). Therefore, therapeutic intervention with PI3K inhibitors appears to be an attractive option for anti-NMDAR encephalitis. In the present study, the potential effects of PI3K inhibitors or agonists on behavioral symptoms were investigated, such as TJ alterations, BBB permeability, and neurological impairment in mice with anti-NMDAR encephalitis. Our results indicate that PI3K inhibition could down-regulate the expression levels of p-PI3K and p-Akt, while up-regulating the expression levels of ZO-1 and Claudin-5. This leads to reducing BBB permeability and improving neurological function in mice with anti-NMDAR encephalitis. Furthermore, PI3K inhibition could play a protective role against the altered BBB function and neuronal damage in mice with anti-NMDAR encephalitis.

Previous studies have suggested that TJs are an important factor in maintaining the integrity of the BBB (Kondoh et al. 2008). At present, it is believed that TJs are composed of a group of molecular protein elements which are the key components for regulating the passage of substances through intercellular connections (Mackic et al. 1999). Among them, Claudin-5 is a specific protein in brain microvascular endothelial cells (Sawada et al. 2003). Studies have shown that Claudin-5 is a necessary and sufficient condition for the formation of TJs (Mitic et al. 2000). In addition, ZO-1 was the first identified TJ-associated protein. Accumulating evidence shows that the loss of ZO-1 leads to the disruption of the TJ structure (Park et al. 2004). In this study, the anti-NMDAR encephalitis mouse model was established by the peptide active immunization method (Ding et al. 2021). The permeability change and BBB destruction in the brain injury of anti-NMDAR encephalitis mice were confirmed by the sodium fluorescein method. The decreased expressions of TJ-related proteins ZO-1 and Claudin-5 were detected by Western blot, qRT-PCR, and immunofluorescence double staining. Interestingly, the decreased expression levels of ZO-1 and Claudin-5 may lead to the destruction of brain microvascular endothelial cell TJs. In this study, the degree of TJ destruction in cerebral microvascular blood endothelial cells was observed by transmission electron microscopy. The results confirmed that the degree of TJ destruction was significantly and negatively correlated with the expression levels of TJ-related proteins ZO-1 and Claudin-5.

PI3K is a lipid kinase that, when activated upstream PI3K, mediates the phosphorylation of its downstream effector Akt (Abbott et al. 2010). Akt is involved in axon guidance, dendritic development, dendritic spine morphogenesis, and synaptic plasticity. It plays an important role in the physiological and pathological processes of the nervous system. In addition, our previous studies have shown that over-activation of NMDAR by NMDA induces decreased expression of TJs in human cerebrovascular endothelial cells (HBEC-5i) and ultimately leads to the destruction of BBB function (Mao et al. 2022; Yu et al. 2022), while the destruction of TJ proteins in cerebrovascular endothelial cells depends on the phosphorylation of PI3K/Akt by MAPKs signaling (Cong and Kong 2020). Notably, modulation of the PI3K/Akt signaling pathway has been shown to be effective in endothelial TJ assembly/disassembly in the current study. A previous study found that the PI3K/Akt signaling pathway is involved in perfluorooctane sulfonate-mediated opening of TJs in brain endothelial cells (Wang et al. 2011). LY294002 is an inhibitor of the PI3K/Akt-signaling pathway, which can prevent Akt phosphorylation induced by 3-chloropropanediol, along with the early and late loss of pericellular Claudin-5 expression in mouse brain endothelial cells. This can decrease the degree of BBB destruction (Camire et al. 2014). Therefore, some peptides, drugs, agonists, and inhibitors may be used to improve endothelial TJ-related diseases. It was surprising to see in the present study that the expression of p-PI3K and p-Akt increased in anti-NMDAR encephalitis mice, while the expression of ZO-1 and Claudin-5 decreased. The increase of BBB permeability may be related to the increase of p-PI3K and p-Akt expression. To further explore the role of PI3K/Akt in BBB destruction caused by anti-NMDAR encephalitis mice, LY294002 (PI3K inhibitor) was used to intervene PI3K. This could reverse BBB damage, along with the degradation of ZO-1 and Claudin-5 in anti-NMDAR encephalitis mice. The decrease of p-PI3K and p-Akt was parallel to the increase of ZO-1 and Claudin-5 expression. However, the treatment with Recilisib (PI3K agonist) further aggravated BBB destruction in anti-NMDAR encephalitis mice. The above results indicate that BBB function damage and the changes of ZO-1 and Claudin-5 in transcription level and protein level in anti-NMDAR encephalitis mice may play a role by activating the phosphorylation of the PI3K/Akt pathway.

Anti-NMDAR encephalitis is a severe neuropsychiatric disease. Previous studies have reported that anti-NMDAR antibodies react with the *N*-terminal domain of the NR1 subunit of NMDAR, causing selective and reversible internalization and degradation of NMDAR. This may lead to lowering or even losing NMDAR-mediated synaptic function and reducing NMDAR-dependent hippocampal long-term potentiation (LTP), thus leading to memory impairment and abnormal mental behavior (Platt et al. 2017). In a previous study, injecting autoantibodies from anti-NMDAR encephalitis patients into the lateral ventricles of mice would result in impaired recognition and memory after 10 days, which were reversible after antibody clearance (Planagumà et al. 2015). In our study, many anti-NMDAR antibodies can be detected in cerebrospinal fluid and serum of anti-NMDAR encephalitis mice, which is consistent with the results of Ding et al. (2021). Moreover, neuron death occurred in the hippocampus of the model. Moreover, the expression of NeuN and MAP-2 protein decreased. In addition, the mice showed short-term and long-term neurological deficits in mNSS evaluation and the Morris water maze test. Interestingly, the protein blot showed that NMDAR on the surface of the hippocampal cell membrane of anti-NMDAR encephalitis mice decreased, which damaged NMDAR function. NMDAR is essential for establishing synaptic plasticity and memory formation, which may explain the memory behavior defects observed in our animal models.

Under normal conditions, the BBB is essential to provide a permissive microenvironment for neuronal function, which largely limits the infiltration of inflammatory cytokines, immune cells, and various exogenous drugs (Selmi et al. 2016). Therefore, the subtle changes of BBB integrity play a vital role in protecting neurons from damage. BBB-targeted research may open new ideas for the treatment of a series of nerve damage caused by NMDAR. In this study, anti-NMDAR encephalitis mice showed obvious neuron loss. However, PI3K inhibitor LY294002 reduced these effects and improved neuronal plasticity, while the PI3K agonist further aggravated neuronal damage. This may be related to PI3K inhibitor LY294002 increasing the expression level of ZO-1 and Claudin-5, effectively improving BBB permeability after brain injury in mice with anti-NMDAR encephalitis and thus reducing the damage of anti-NMDAR antibody to NMDAR. Notably, the PI3K/Akt pathway can affect brain development and various nervous system diseases by regulating the survival, growth, polarity, synaptic plasticity, and circuits of neurons. For example, the PI3K/Akt pathway can induce the synthesis of local proteins such as the NMDAR NR1 subunit, affecting long-term enhancement LTP and long-term inhibition LTD. This can also regulate synaptic plasticity, which in turn affects the learning and memory function of the brain. However, PI3K inhibitor LY294002 prevents NMDAR-LTD induced by dihydroxyphenylglycine (DHPG) (Hou and Klann 2004). Our data further show that PI3K inhibition can significantly reduce NMDAR internalization and neuronal loss, improving the neurobehavior deficit of anti-NMDAR encephalitis mice.

At present, the pathogenesis of autoimmune diseases is still unclear. However, a variety of regulatory factors have been identified, including external and internal environmental triggers such as hormones and microbiome, genetics, epigenetics (miRNA, histone deacetylation and methylation), infection, and so on (Khan and Ansar Ahmed 2015). Clinically, anti-NMDAR encephalitis is more common in women, with previous reports in the United States and Europe showing that more than 80% of patients are female and are usually associated with ovarian teratoma (Titulaer et al. 2013). This feature has also been noted in many animal models of autoimmune diseases, where female mice may be more susceptible to symptoms compared to males (Ansar Ahmed et al. 1985). Studies have shown that female mice are often used to construct a variety of autoimmune disease models (Ding et al. 2021; Linnoila et al. 2019). Therefore, in this study, female C57BL/6J mice were used to establish an anti-NMDAR encephalitis mouse model.

In conclusion, the results of this study indicate that the TJ-associated proteins ZO-1 and Claudin-5 are closely related to BBB destruction in anti-NMDAR encephalitis mice. Interestingly, LY294002 can prevent the reduction of TJ-related proteins ZO-1 and Claudin-5, protect the BBB, reverse the reversible loss of NMDAR, reduce neuronal damage, and thus relieve neurobehavior symptoms. However, the specific mechanism of BBB destruction and PI3K inhibition on the expression level of TJ-related proteins ZO-1 and Claudin-5 in mice with anti-NMDAR encephalitis need to be further studied. Whether the expression of other TJ-related proteins changes at the same time also provides an area for further research. Targeting the endothelial TJ-related signaling pathway to protect BBB integrity and restore its physiological function in the early stages of the disease will contribute to the prevention, control, and eventual reversal of the neurodegenerative process. In turn, this provides great potential for improving the clinical prognosis of BBB-related neurological diseases in the future.

## Supplementary Information

Below is the link to the electronic supplementary material.

Supplementary file1 (DOCX 16 KB)

## Data Availability

Enquiries about data availability should be directed to the authors.
