# Supplementary material for: Inhibiting PI3K/Akt-Signaling Pathway Improves Neurobehavior Changes in Anti-NMDAR Encephalitis Mice by Ameliorating Blood–Brain Barrier Disruption and Neuronal Damage
Source: Cell Mol Neurobiol. 2023 Jun 14;43(7):3623–37. doi: 10.1007/s10571-023-01371-3 (PMC10477152; doi:10.1007/s10571-023-01371-3)
Supplement: Supplementary file 1 — Supplementary file1 (DOCX 16 KB) [file 10571_2023_1371_MOESM1_ESM.docx]

Table1 modified neurologic severity score (mNSS)

| item | Performance | Scores/point |
| --- | --- | --- |
| Tail lifting experiment  (3 points) | Forelimb flexion | 1 |
|  | Hindlimb flexion | 1 |
|  | The head deviates from the vertical axis more than 10 ° in 30 seconds. | 1 |
| Walking experiment  (3 points) | Normal walking | 0 |
|  | Unable to walk normally | 1 |
|  | Turn to the side of the paralysis | 2 |
|  | Pour to the side of the paralysis | 3 |
| Sensory experiment  (2 points) | Placement experiments (visual and tactile tests) | 1 |
|  | Proprioceptive experiment (pressing mouse claws to the edge of the table to stimulate limb muscles) | 1 |
| Balance beam experiment  (6 points) | Stable balance posture | 0 |
|  | Hold on to the edge of the balance beam | 1 |
|  | Cling to the balance beam, one side of the limb slips | 2 |
|  | Cling to the balance beam, the both limbs fall or rotate from the balance beam (> 60 s) | 3 |
|  | Try to balance on the balance beam but fall (> 60 s) | 4 |
|  | Try to balance on the balance beam but fall (> 20 s) | 5 |
|  | Fall, no attempt to balance on the balance beam (< 20 s) | 6 |
| Reflection and abnormal activity  (4 points) | Auricular reflex (shake the head when touching the external auditory canal) | 1 |
|  | Corneal reflex (blink when touching the cornea with cotton silk) | 1 |
|  | Panic reflex (movement reflex or scream after hearing a sudden sound) | 1 |
|  | Epilepsy, myoclonus, dystonia | 1 |
| Total score |  | 18 |
